# Evaluating the association between feed efficiency and the fecal microbiota of early-life Duroc pigs using 16S rRNA sequencing

**DOI:** 10.1186/s13568-020-01050-2

**Published:** 2020-06-19

**Authors:** Jinglei Si, Lingli Feng, Jiuyu Gao, Ye Huang, Guangjie Zhang, Jiayuan Mo, Siran Zhu, Wenjing Qi, Jing Liang, Ganqiu Lan

**Affiliations:** College of Animal Science & Technology, Guangxi University, Nanning, 530004 China

**Keywords:** 16S rRNA, Fecal microbiota, Feed efficiency, High-throughput sequencing, Pig, Residual feed intake

## Abstract

Improving the predication efficiency of porcine production performance at early stage will contribute to reducing the breeding and production costs. The intestinal microbiota had received plenty of attention in recent years due to their influence on host health and performance. The purpose of this study was to investigate the relationship between the fecal microbiota at early growth period and porcine feed efficiency (FE) under a commercial feeding environment. Ninety-one pigs were reordered according to the residual feed intake (RFI) values between day 90 on test and day 160 off test, 9 lowest RFI pigs and 9 highest RFI pigs were selected as the LRFI group and the HRFI group, respectively. Fecal samples from pigs in the early grower phase (day 80) were performed for microbial diversity, composition, and predicted functionality by using 16S rRNA sequencing. The results showed that no significant differences in microbial alpha diversity were observed between two RFI groups, whereas, some RFI-associated compositional differences were revealed. In particular, the microbiota of the LRFI group (more feed-efficient) had significantly higher levels of some members of *Clostridiales* and *Bacteroidales* (e.g., *g_1_68* and *g_norank_f_p_2534_18B5*), which may promoted FE through protecting gut barrier function, compared with those of the HRFI pigs. Kyoto Encyclopedia of Genes and Genomes (KEGG) pathways analysis found that the LRFI pigs were likely have microbiota with higher levels of amino acid metabolism. Moreover, redundancy analysis (RDA) showed that litter size, parity, and date of birth had significant effects on the bacterial community structure. These results improved our knowledge of the porcine early-life fecal microbiota and its potential link underlying RFI, which would be useful for future development of microbial biomarkers for predicting and improving porcine FE as well as investigation of targets for dietary strategies.

## Introduction

Feed accounts for more than 60% of total production costs in growing pigs. Therefore, improving FE has been an important part of the breeding goal in commercial pig production for both economic and environmental reasons. A variety of factors that can influence porcine FE, such as genetics (Do et al. 2014; Onteru et al. 2013; Reyer et al. 2017), diseases (Patience et al. 2015), environment (Chatelet et al. 2018), and diets (Collins et al. 2017; Gilbert et al. 2018). RFI, defined as the difference between the observed feed intake and predicted feed requirements based on average daily gain and backfat, is a useful criterion to measure net FE based on the biological mechanisms that influence FE (Gilbert et al. 2017; Herd and Arthur 2009). Intestinal microbiota is considered as a major “factor”, which plays an important role in the processing of nutrients and the acquisition of energy (Fouhse et al. 2016; Ramayo-Caldas et al. 2016; Xiao et al. 2016). The contribution of intestinal microbiota to pig health and performance, including digestion and metabolism of nutrients, stimulation of immune response, protection from pathogens and stimulation of epithelium cell proliferation is becoming increasingly apparent (Katouli et al. 1997; Konstantinov et al. 2006; Mann et al. 2014; Spreeuwenberg et al. 2001; Thompson et al. 2008). So, the intestinal microbiota could potentially be used to predict or improve porcine FE.

In recent years, the correlations between microbial community and phenotypic traits have become the focus of much attention (Buzoianu et al. 2012; Frese et al. 2015; Pedersen et al. 2013; Vigors et al. 2016). Many researchers have found FE-related bacterial groups in pigs (Quan et al. 2018; Tan et al. 2018; Yang et al. 2017), chickens (Siegerstetter et al. 2017; Yan et al. 2017), and cattle (Li et al. 2019). In pigs, the early colonization and succession of intestinal microbiome has been found to be important for the formation of host phenotypes and specific microbial composition (Collins et al. 2017; Han et al. 2017; Tian et al. 2017). However, little is known about whether the composition of fecal microbiome in young pigs might be used to predict the phenotype of grower-finisher hosts. It had been reported that the increase of *Firmicutes* with pigs’ growth was consistent with the significantly increased fat deposition in older pigs, compared to 1 month old piglets (Zhao et al. 2015). A large-scale study showed that the fecal microbiota diversity at week 15 and end-of-feeding stage were strongly correlated with back fat and average daily gain of crossbred pigs, whereas very low correlation were detected at weaning (Lu et al. 2018). In the current study, we aimed to investigate the possible relationship between the fecal microbiota and RFI trait of growing stage pigs under a commercial environment. For the purpose, we compared the composition and potential functionality of the fecal microbiota in LRFI and HRFI Duroc pigs using 16S rRNA gene sequencing, and analyzed the effects of host and environmental factors on the microbial community structure.

## Materials and methods

### Animals management and sample collection

Ninety-one purebred Duroc pigs used in this study were raised in a commercial farm (Nanning, Guangxi, China). The piglets were weaned at the same age of 28 days and raised under the same nursery conditions. All experimental pigs were moved to an environmentally controlled fattening house (ten pigs in each pen) at the age of day 70, and were fed with the same standard diets without antibiotics or medicines. Daily feed intake (DFI) and individual body weights (BW) data were recorded by Electronic Feed Intake Recording Equipment (FIRE, Osborne, USA) and used to calculate performance indicators, such as average daily feed intake (ADFI), average daily gain (ADG), and feed conversion ratio (FCR). ADFI, individual BW, and FCR data were collected from day 90 to 160. The backfat thickness (BF) was measured using ultrasound measurements (Corometrics Medical Systems, Inc., Wallingford, CT, USA). The equation used to predict RFI has been previously described (Cai et al. 2008). Fresh fecal samples were collected from each individual at 80 ± 1.15 days old and kept frozen in liquid nitrogen for transportation, and then stored at − 80 °C until use.

### DNA extraction and sequencing

The DNA extraction, 16S rRNA gene PCR, library preparation and DNA sequencing of the fecal samples were performed by a commercial provider (Shanghai Majorbio Bio-pharm Technology Co.,Ltd, China). The V3–V4 region of the 16S rRNA gene was amplified by polymerase chain reaction (PCR) with universal bacterial 16S rRNA gene PCR amplicon primers (341F-806R) (Kozich et al. 2013). According to the preliminary quantitative results of electrophoresis, the PCR products were detected and quantified by quantifluor™-St blue fluorescence quantitative system (Promega company), and then mixed in corresponding proportion according to the requirements of the sequencing quantity of each sample. All sample libraries sequencing were performed on the Illumina MiSeq platform (Illumina, USA).

### Microbial analysis

The 16S rRNA sequencing data were processed using the Quantitative Insights Into Microbial Ecology (QIIME1) (version 1.9.1) platform (Caporaso et al. 2010). The raw sequence reads were filtrated with a minimum overlap of 10 bp and a maximum mismatch ratio 0.2 by using FLASH (version 1.2.11). Operational taxonomic units (OTUs) were picked at 97% similarity cut-off, and the identified taxonomy was then aligned using the Greengenes database (version 13.8). Chimeric sequences were identified and removed in the process of clustering with the software of USEARCH (version 7). OTUs with number of sequences < 20 of the total number of sequences were removed from the OTU table with the software of USEARCH.

The microbial alpha diversity indices of the samples were determined using the Chao1 (richness estimator) (Chao 1984), ACE (abundance-based coverage estimator) (Chao and Yang 1993), Sobs (the observed richness), Shannon (entropy estimator) (Shannon 1948), Simpson (Simpson’s index calculator) (Simpson 1949), Coverage (community coverage), Shen, and PD (Phylogenetic diversity) (Faith 1992), and these indices analyses were also calculated within Mothur (version 1.30.2). Principal component analysis (PCA) was performed at the phylum and the genus level, and the results were visualized using the STAMP program (version 2.1.3) (Parks et al. 2014). Linear discriminant analysis (LDA) effect size (LEfSe) were performed using the LEfSe tool (Segata et al. 2011). The OTU functions of fecal microbial were predicted by the KEGG database, based on performed using phylogenetic investigation of community by reconstruction of unobserved States (PICRUSt) (Langille et al. 2013). RDA was executed, and the significance of total host and environmental factors (including pen, season, birth weight, parity, date of birth, litter size) was tested with Monte Carlo permutations (permu = 999). Host and environmental factors were selected by the functions of envfit (permu = 999) and vif, and the factors with P > 0.05 or vif > 10 were removed from the following analysis.

## Result

### Basic statistics of porcine performance and RFI

The average RFI value of LRFI and HRFI was − 0.047 ± 0.11 (mean ± SD) (Table 1). Compared with HRFI pigs, ADFI of LRFI pigs was lower 0.45 kg/day (P < 0.01), but showed an improvement in FCR of 0.42 (P < 0.01). However, no remarkable differences between the HRFI and LRFI were observed for ADG (P > 0.05), 100 kg BF (P > 0.05), day 90 BW (P > 0.05), day 160 BW (P > 0.05) and other phenotypes (P > 0.05) (Additional file 1). The phenotypic distributions of 91 pigs including ADFI, ADG, FCR, 90d BW, 160d BW, 100 kg BF and RFI values in the experimental cohort were shown in Additional file 1.

**Table 1:** Effect of ranking pigs by RFI on growth performance parameters

Parameter	HRFI (n = 9)	LRFI (n = 9)	P-value^b^
RFI	0.13 ± 0.050	− 0.23 ± 0.10	< 0.001***
FCR	2.62 ± 0.19	2.20 ± 0.17	< 0.001***
ADFI (kg/day)	2.67 ± 0.31	2.22 ± 0.26	< 0.001***
ADG (kg/day)	1.02 ± 0.12	1.01 ± 0.070	0.86
100 kg BF (cm)	12.82 ± 2.08	11.17 ± 2.28	0.13
90 d BW (kg)	44.44 ± 8.39	41.20 ± 7.51	0.40
160 d BW (kg)	114.16 ± 13.45	110.11 ± 10.60	0.41

The data were expressed as the mean values ± standard deviation (SD)

*RFI* residual feed intake, *FCR* feed conversion ratio, *ADFI* average daily feed intake, *ADG* average daily gain, *100* *kg BF* 100 kg back fat thickness, *90* *d BW* 90 day body weight, *160* *d BW* 160 day body weight

^b^The P values were determined using Welch’s t test (*P < 0.05; **P < 0.01; ***P < 0.001)

### Differences in the fecal bacterial alpha diversity of the HRFI and LRFI pigs

A total of 3,254,159 sequence reads were obtained from 91 fecal samples, with an average of 35,759 reads per sample (ranging from 23,641 to 73,941). After subsampling each sample to an equal sequencing depth (23,562 reads per sample) and clustering, 1011 OTUs at 97% identity were obtained. From a taxonomic perspective, 14 phyla, 24 class, 33 order, 60 families, and 114 genera were identified across all pig fecal samples.

Sobs, Shannon index, Simpson index, ACE, Chao 1, Coverage, Shen, PD index values were used as parameters of the alpha diversity of fecal microbiota in our study (Table 2). The Sobs metric was 575.33 for the LRFI group and 549.22 for the HRFI group. The Shannon metric was 4.48 for the LRFI group and 4.32 for the HRFI group. The Simpson metric was 0.03 for the LRFI group and 0.06 for the HRFI group. The ACE metric was 660.52 for the LRFI group and 637.15 for the HRFI group. The Chao 1 metric was 670.15 for the LRFI group and 644.95 for the HRFI group. The Coverage metric was 0.99 for the LRFI group and 0.99 for the HRFI group. The Shen metric was 62.50 for the LRFI group and 64.95 for the HRFI group. The PD metric was 48.09 for the LRFI group and 45.48 for the HRFI group. No significant differences for any of the indices of alpha diversity measured (P > 0.05).

**Table 2:** Differences in the fecal microbial diversity of the two groups

Estimators	HRFI-mean ± SD	LRFI-mean ± SD	P-value^a^
Sobs	549.22 ± 86.52	575.33 ± 59.45	0.47
Shannon	4.32 ± 0.67	4.48 ± 0.27	0.53
Simpson	0.06 ± 0.080	0.03 ± 0.010	0.30
Ace	637.15 ± 72.74	660.52 ± 61.97	0.47
Chao	644.95 ± 66.64	670.15 ± 67.06	0.44
Coverage	0.99 ± 0.00	0.99 ± 0.00	0.42
Shen	64.95 ± 8.58	62.50 ± 8.49	0.92
pd	45.48 ± 5.34	48.09 ± 3.99	0.26

The data were expressed as the mean values ± standard deviation (SD)

^a^The P values were determined using Welch’s t test (*P < 0.05; **P < 0.01; ***P < 0.001)

### Differences in the fecal microbial taxa represented in the HRFI and LRFI pigs

**Fig. 1:**
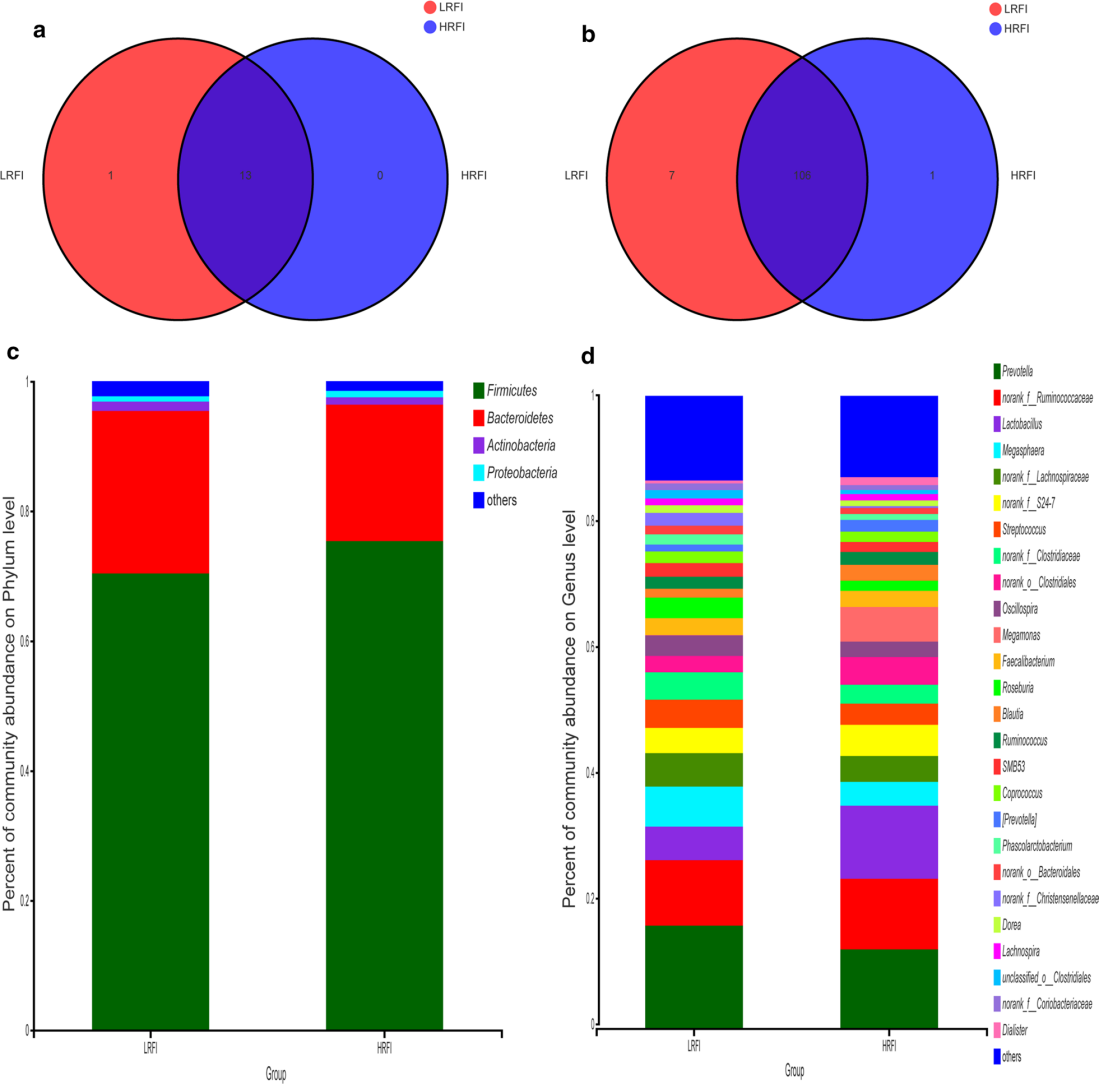
Compositions of the fecal microbiota of the LRFI and HRFI pigs. The number of phylum (**a**) and genera (**b**) shared by the two groups are shown in Venn diagrams. The different colors represent different groups, the numbers in the overlapping part represent the number of species in two groups, and the numbers in the non-overlapping parts represent the number of species unique to the corresponding group. The overall compositions of the fecal microbiota of the LRFI and HRFI groups were represented as bar plots at the phyla (**c**) and the genus level (**d**). The different colors represent the columns, and the length of the columns represent the proportion of the species in the two groups

At the phylum level, the microbiota of the LRFI and HRFI groups shared 13 phyla (92.86%, Fig. 1a), whereas *Fusobacteria* uniquely identified in the LRFI group. The three dominant phyla detected in both groups were *Firmicutes* (70.41% in the LRFI group and 75.34% in the HRFI group), *Bacteroidetes* (25.02% in the LRFI group and 21.04% in the HRFI group), and *Actinobacteria* (1.43% in the LRFI group and 1.13% in the HRFI group) (Fig. 1c).

At the genus level, the fecal microbiota of the two groups shared 106 genera (92.98%), with 7 (6.14%) and 1 genus (0.0088%) uniquely identified in the LRFI group and the HRFI group, respectively (see Fig. 1b). Four dominant genera, *Prevotella* (16.25%), *norank_f_Ruminococcaceae* (10.25%), *Megasphaera* (6.34%), and *norank_f_Lachnospiraceae* (5.31%) were found in the LRFI group, whereas the four dominant genera in the HRFI group were *Prevotella* (12.48%), *Lactobacillus* (11.57%), *norank_f_Ruminococcaceae* (11.18%), and *norank_f_S24*-*7* (4.93%) (Fig. 1d).

**Fig. 2:**
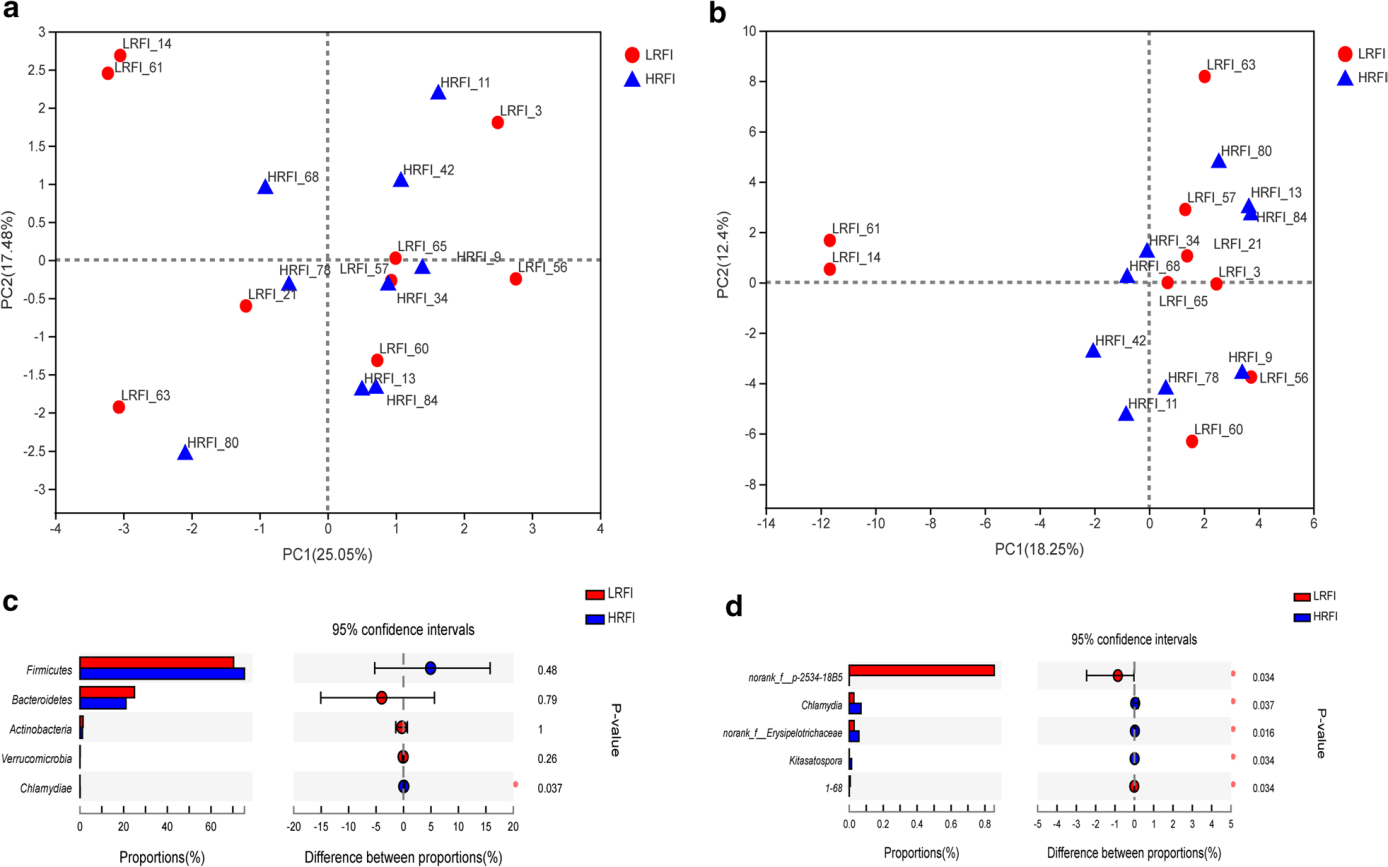
Composition of the fecal microbiota of the LRFI and HRFI pigs at the phylum and genera level. Principal component analysis (PCA) plot at phylum (**a**) and genera (**b**) level. The X-axis and Y-axis represent the principal component axes, and the percentage represents the value of the component’s interpretation of the different sample composition. The points of different colors or shapes represent samples of different groups, and the closer the two sample points, the more similar the two species composition. The phylum (**c**) and genera (**d**) represented at significantly different levels in the microbiota of two groups were shown in an extended error bar plot. The X-axis represents different groups, different colored boxes represent different groups, and the Y-axis represents the average relative abundance of a species in different groups. The differences in the compositions were tested using a two-sided Welch’s test, and P < 0.05 was marked with “*”

PCA was used to compare the total microbial composition of the LRFI and HRFI pigs at the level of phylum and genus. In the PCA diagram, the microbial communities were not separated at phylum (Fig. 2a) and genus (Fig. 2b) levels. Then, we compared the relative abundance of microbial members between the two groups and found some significant different phyla and genera (P < 0.05), although they have a lower abundance. Such as the level of *Chlamydiae* phylum was significantly higher in the HRFI group than those in the LRFI group (P = 0.037) (Fig. 2c). At the genus level, the levels of *Chlamydia* (P = 0.0034), *norank_f__Erysipelotrichaceae* (P = 0.016), *g_Kitasatospora* (P = 0.0034) were significantly higher in the HRFI group. In contrast, the levels of *norank_f_p_2534_18B5* (P = 0.034), *g_1_68* (P = 0.034) were significantly higher in the LRFI group than those in the HRFI group (Fig. 2d).

**Fig. 3:**
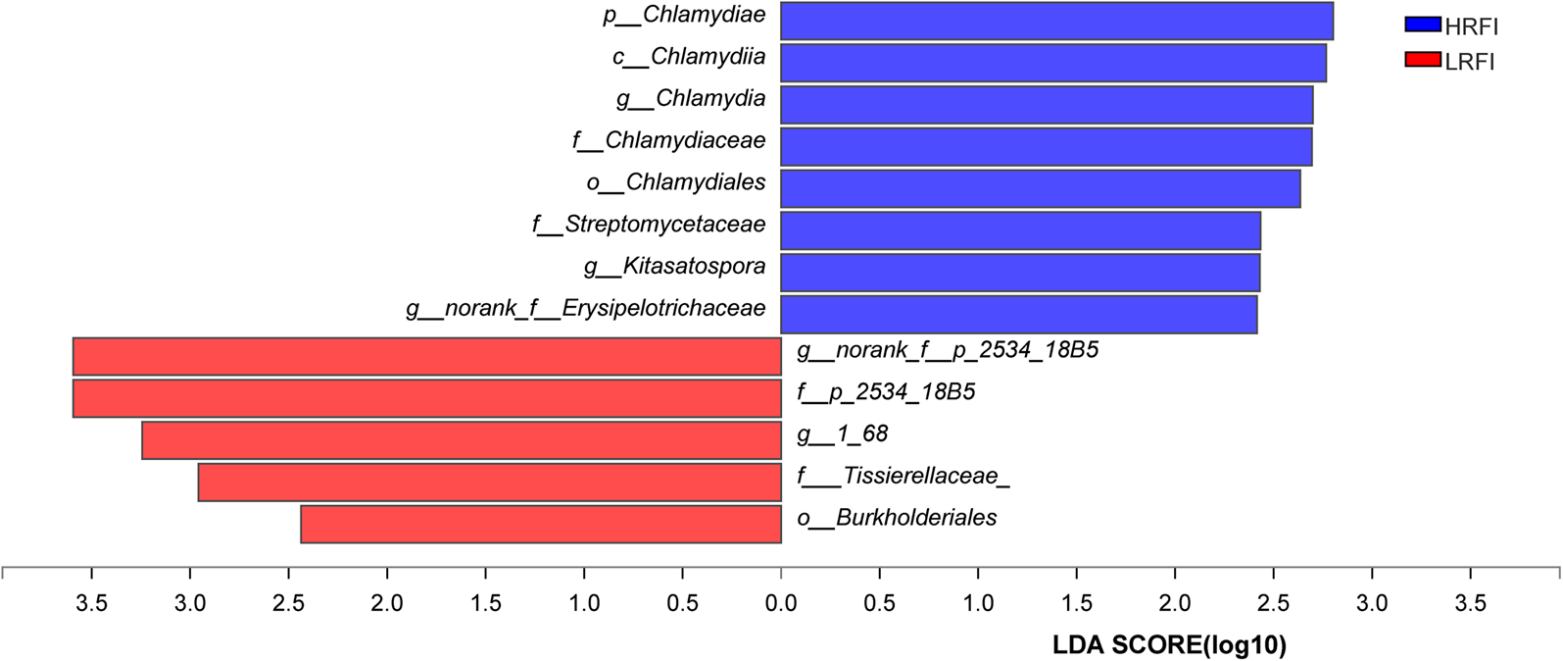
Indicator bacteria with LDA scores of 2 or greater in bacterial communities associated with LRFI and HRFI groups. The X-axis represents the LDA score, different colored boxes represent different groups, and the higher the LDA score, the greater the impact of species abundance on the difference effect

The LEfSe tool was used to identify specialized microbial communities in the HRFI and LRFI groups. The results of LEfSe analysis showed that 5 (3 genera unique to the HRFI group and 2 genera unique to the LRFI group) genera were potential biomarkers for distinguishing between high and low RFI groups (Fig. 3). These results confirmed the significant of enrichment of *p_Chlamydiae*, *c_Chlamydiia*, *g_Chlamydia*, *f_Chlamydiaceae*, *o_Chlamydiales*, *f_Streptomycetaceae*, *g_Kitasatospora* and *norank_f__Erysipelotrichaceae* in the HRFI group. We also found that, *o_Burkholderiales*, *f_Tissierellaceae_*, *g_1_68*, *p_2534_18B5*, and *norank_f_p_2534_18B5* were higher relative abundances in the LRFI group compared to those in the HRFI group (Fig. 3).

### Predicted KEGG pathways differences of fecal microbiota between the HRFI and LRFI pigs

**Fig. 4:**
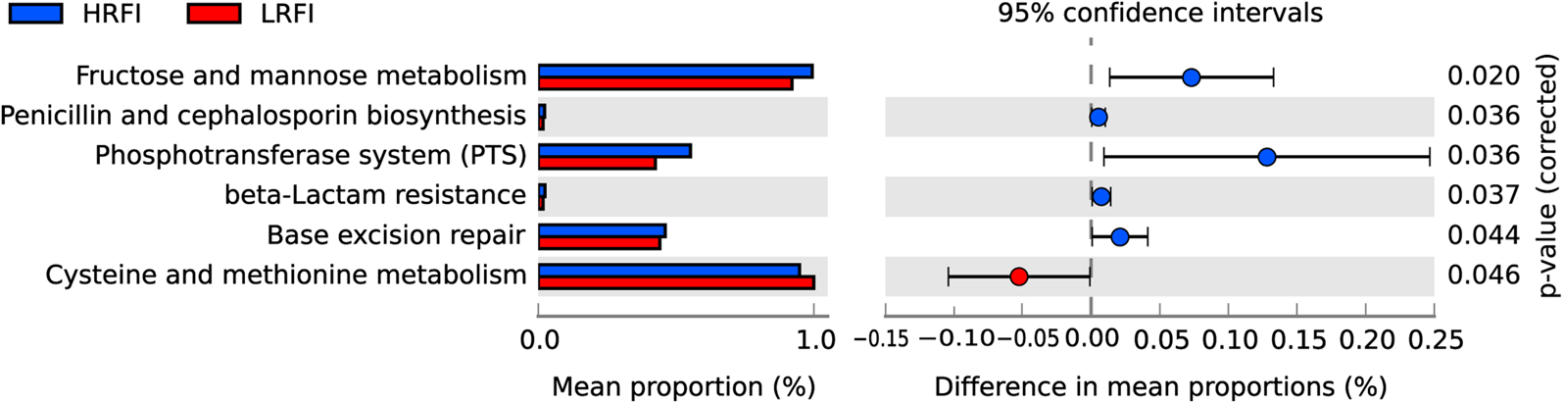
The different functions of the fecal microbiota of the LRFI and HRFI groups. The microbial functions were predicted using PICRUSt at the third level of the KEGG pathway and were expressed as relative. The differences in the compositions were tested using a two-sided Welch’s test, permutation test replicates 1000, and P < 0.05 was considered significant

Several of the predicted KEGG pathways were significantly differentially identified in the fecal microbiota of the HRFI and LRFI pigs (Fig. 4). “Cysteine and methionine metabolism” pathway was predicted at significantly a higher level in the microbiota of the LRFI group (1.00%) than in that of the HRFI group (0.95%). In contrast, “fructose and mannose metabolism”, “penicillin and cephalosporin biosynthesis”, “phosphotransferase system (PTS)”, “beta-Lactam resistance”, and “base excision repair” pathways which were involved in “carbohydrate metabolism”, “biosynthesis of other secondary metabolites”, “membrane transport”, “drug resistance: antimicrobial”, and “replication and repair” at the higher KEGG pathway hierarchical levels, were predicted at significantly higher levels in the microbiota of the HRFI group (0.99, 0.02, 0.55, and 0.02%, respectively) than those in that of the LRFI group (0.92, 0.02, 0.43, and 0.02%, respectively).

### Host and environmental factors affecting porcine fecal microbial community structure

**Fig. 5:**
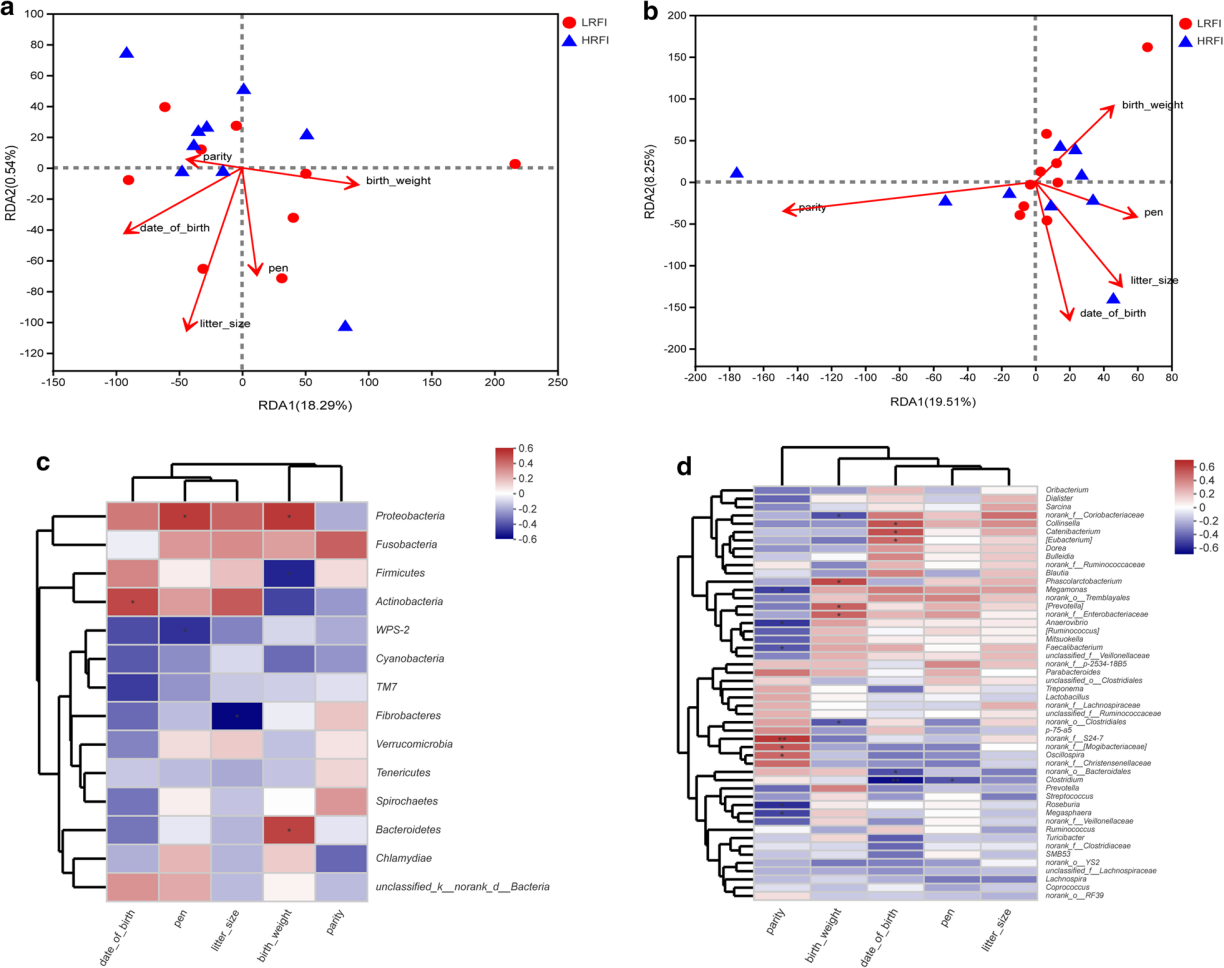
The relationship of host and environmental factors to the microbial community structure. At phylum (**a**) and genus (**b**) level Distance-based Redundancy Analysis (RDA) plot showing the relationship of pen, season, birth weight, parity, date of birth and litter size to the microbial community structure. The red arrow indicates the host and environmental factors. The length of the arrow was representing the degree of interpretation of the factors to the species. The angle between the arrows represents the correlation. The distance from the sample point to the factor represents the relative influence of the factor on the distribution of the microbial community. Correlation heatmap of the top fourteen phyla (**c**), the top fifty genus (**d**) and environmental and host factors. The X-axis and Y-axis were host and environmental factors and species, respectively. The R-value was shown in different colors in the figure. The legend on the right was the color range of different R values. The value of P < 0.05 or P < 0.01 was marked with “*” or “**”

Host and environmental factors may influence microbial community structures. Therefore, we investigated the possible correlations between the microbial community structure and the variables of environmental and host characteristics. After removal of the redundant variables, five host and environmental characteristics (including pen, birth weight, parity, date of birth, and litter size) were chosen for RDA. As a result, litter size was found to have significant (P = 0.007) effect on the fecal bacterial community structure at phylum level (Fig. 5a), which the first axis and second axis explained 21.15% and 0.56% of total microbial variance, respectively. Besides, at genera level, date of birth and parity had significant (P = 0.019 and 0.024) effects on the fecal microbial composition (Fig. 5b), which the first axis and second axis explained 21.17% and 8.32% of total microbial variance, respectively.

The correlation heatmap showed that the relationship between bacterial phyla and environmental factors were different (Fig. 5c). *Actinobacteria* showed a significant positive correlation with date of birth (Spearman correlation coefficient: 0.50, P = 0.036). *Fibrobacteres* demonstrated a significant negative correlation with litter size (Spearman correlation coefficient: − 0.59, P = 0.010). The factor of pen was significant positive correlated with *Proteobacteria* (Spearman correlation coefficient: 0.53, P = 0.020), whereas significant negative correlated with *WPS*-*2* (Spearman correlation coefficient: − 0.49, P = 0.040). The birth weight revealed a significant negative correlation with *Firmicutes* (Spearman correlation coefficient: − 0.52, P = 0.027), whereas significant positive correlated with *Proteobacteria* (Spearman correlation coefficient: 0.54, P = 0.021) and *Bacteroidetes* (Spearman correlation coefficient: 0.51, P = 0.032). Similarly, result of heatmap showed the relationship between environmental factors and bacterial genera at the genus level were different (Fig. 5d). The factor of date of birth was significant positive correlated with [*Eubacterium*] (Spearman correlation coefficient: 0.47, P = 0.049), *Catenibacterium* (Spearman correlation coefficient: 0.55, P = 0.018), *Collinsella* (Spearman correlation coefficient: 0.54, P = 0.021) and *g_Oscillospira* (Spearman correlation coefficient: 0.48, P = 0.044), whereas significant negatively correlated with *Clostridium* (Spearman correlation coefficient: − 0.66, P = 0.003), *g_norank_o_Bacteroidales* (Spearman correlation coefficient: − 0.49, P = 0.038), *g_norank_o_Clostridiales* (Spearman correlation coefficient: − 0.47, P = 0.049). The birth weight revealed a significant positive correlation with [*Prevotella*] (Spearman correlation coefficient: 0.49, P = 0.036), *Enterobacteriaceae* (Spearman correlation coefficient: 0.49, P = 0.039) and *Phascolarctobacterium* (Spearman correlation coefficient: 0.58, P = 0.012), nevertheless, significant negatively correlated with *g_norank_f_Coriobacteriaceae* (Spearman correlation coefficient: − 0.49, P = 0.041). The factor of parity was significant positive correlated with [*Mogibacteriaceae*] (Spearman correlation coefficient: 0.53, P = 0.024) and *g_norank_f_S24*-*7* (Spearman correlation coefficient: 0.63, P = 0.005), however, significant negatively correlated with *Roseburia* (Spearman correlation coefficient: − 0.58, P = 0.012), *Anaerovibrio* (Spearman correlation coefficient: − 0.55, P = 0.017), *Faecalibacterium* (Spearman correlation coefficient: − 0.47, P = 0.049). *Megamonas* (Spearman correlation coefficient: − 0.53, P = 0.024) and *Megasphaera* (Spearman correlation coefficient: − 0.52, P = 0.026). The factor of pen was significant positive correlated with *Clostridium* (Spearman correlation coefficient: − 0.48, P = 0.044).

## Discussion

Recent studies in porcine intestinal microbiome using next-generation sequencing have greatly expanded our understanding on the role of the gut microbiota in different phenotypes (Han et al. 2017; McCormack et al. 2017; Tan et al. 2018), and demonstrated associations between porcine intestinal microbes composition and growth traits. Several studies indicated that the differences within the gut microbiome explained the variability of FE in pigs. For example, *Oscilibacter*, *Christensenellaceae*, and *Cellulosilyticum* were more abundant in high FE pigs (McCormack et al. 2017). In another study, *Ruminococcaceae*, *Christensenellaceae*, *Akkermansia*, and *Lachnospiraceae* were reported to have a positive relationship to porcine FE, nevertheless, *Faecalibacterium* has a negative association with porcine FE (Yang et al. 2017). However, most of these studies were focused on the grower-finisher pigs (Fang et al. 2017; Tan et al. 2018), with very few studies involving the FE of pigs through differences in microbiome comparison of early-life pigs. Bacterial colonization in the gastrointestinal tract of early-life pigs has been suggested to be crucial for the formation of host phenotypes, and concurrently helpful for early selection of pigs (Mach et al. 2015). Early selection of breeding stock can expand the scale of testing and increase selection intensity, especially for low-heritability traits (such as RFI), and can achieve rapid genetic progress.

Consistent with a previous report (Yang et al. 2018), the LRFI pigs tended to have higher α variety (Chao 1, Sob, Shannon, ACE, Coverage, and PD) than the HRFI pigs, albeit no significant difference in richness or evenness were found between the two groups. The composition of intestinal microorganisms can be influenced by diet, age, host genetics and environmental factors (Benson et al. 2010; Buzoianu et al. 2012; Kumar et al. 2019). Previous studies indicated that there were significant differences in intestinal microbial composition among pigs at different grow stages (Ke et al. 2019; Kumar et al. 2019). In this study, the dominant phyla within the fecal microbiota were *Firmicutes*, *Bacteroidetes* and *Actinobacteria*, and the six most abundant genera were *Prevotella*, *Ruminococcaceae_f_norank*, *Lactobacillus*, *Megasphaera*, *Lachnospiraceae_f_norank* and *S24*-*7_f_norank*, although the proportion of each phylum and genera were fluctuant (Camarinha-Silva et al. 2017; Han et al. 2017). Wang et al. reported that *Firmicutes*, *Bacteroidetes*, and *Actinobacteria* were the three most abundant phyla across each stage in the life of a pig, and these microbiomes were defined as core bacteria (Wang et al. 2019). At the genus level, *Prevotella*, *Ruminococcaceae*, *Lactobacillus* were reported as the core bacteria for fecal samples at the ages of 80, 120 and 240 days (Ke et al. 2019).

Specific fecal microbiota could potentially be linked with porcine FE. LEFse analysis found that *g_1_68* in the LRFI pigs, which within the *Clostridiales* order, was significantly higher than in the HRFI pigs. As previously reported (McCormack et al. 2017), most of the RFI-specific OTUs were from the *Clostridiales* order. *Clostridiales* are butyric acid-producing bacteria, which can promote the functional recovery of intestinal mucosa and may inhibit the formation of inflammatory cytokines, thereby play an anti-inflammatory role in the gastrointestinal tract (Augenlicht et al. 2002; Segain et al. 2000). Moreover, the uncultured *p_2534_18B5*, is a member of *Bacteroidales*, which has been proved to have epithelial barrier function in colonic through regulation the secretion of cytokines (Kuhn et al. 2018). In addition, corresponding to the LRFI group, *Chlamydia*, *Kitasatospora*, and uncultured *Erysipelotrichaceae* had a higher abundance in the HRFI group, which were potential pathogenicity and may be the factors that affect porcine FE.

The involvement of different metabolic pathways in the microbiota could further justify differences in the porcine phenotype. In this study, several different KEGG pathways were observed in the LRFI and HRFI groups. “Cysteine and methionine metabolism” related genes were more abundant in the fecal microbiota of the LRFI group than those in that of the HRFI group. Several study pointed out that intestinal bacteria may affect the utilize dietary protein by producing short chain fatty acids (SCFA) and regulating the metabolism of acids (Holzer 2016). We observed that five KEGG pathways (“fructose and mannose metabolism”, “penicillin and cephalosporin biosynthesis”, “phosphotransferase system (PTS)”, “beta-Lactam resistance”, and “base excision repair”) were significantly more abundant in the microbiota of the HRFI group than those in that of the LRFI group, consistent with the results of other studies. For instance, previous studies indicated that PTS is a complex translocation system, which transports sugar to cells and phosphorylates substrates during transport by phosphotransferase (Lengeler et al. 1990). This may reduce the utilization of sugar by the host to some extent. “Penicillin and cephalosporin biosynthesis” and “beta-Lactam resistance”-related genes were more abundant in the fecal microbiota of the HRFI than those in that of the LRFI group. Penicillin and cephalosporin are synthesized by a series of enzymatic reactions that form the tripeptide δ-(l-α-aminoadipyl)-l-cysteinyl-d-valine and convert this tripeptide into the final penicillin or cephalosporin molecules (Martín et al. 1999). Interesting, we observed a significant increase *Chlamydiae* in the HRFI group. Demonstrably, a higher relative abundance of potentially disadvantageous bacteria, such as *Chlamydiae* and *Bacteroidales*, were found in pigs with lower FE. These results indicated that pigs that are more feed efficient are likely to have fecal microbiota with higher levels of amino acid metabolism.

Even though many studies have involved the relationship between microorganisms and FE, few FE-related microbial classifications were found, which may be due to the diet, host genetics and management strategies used in different feeding environments. In the present study, we found that the composition of fecal bacterial community was related to host (especially litter size and parity) and some environment factors. For instance, at the phylum and genus levels, the date of number, litter size and parity have a significant influence on intestinal microbial structure composition. Yang et al. reported that significant effects of sex and kinship on fecal microbial community structure (Yang et al. 2017). However, several studies have suggested no significant correlation between microbiota composition and pen or sex (Mach et al. 2015). The relationship between host or environment factors (such as pen, birth weight, parity, date of birth and litter size) and porcine fecal microbial community structure remains controversial issues. In this study, *Firmicutes* showed a significant negative correlation with birth weight, whereas *Bacteroidetes* has a significant positive correlation with birth weight. Ding et al. reported that the abundances of *Firmicutes* showed a significant negative correlation with pre-weaned weight gain and *Bacteroidetes* showed a significant positive correlation with pre-weaned weight gain in the colon (Ding et al. 2019). However, Han et al. reported that at the phylum level, the microbe of heavier weaned piglets had significantly higher levels of *Firmicutes* and a higher *Firmicutes*-to-*Bacteroidetes* ratio than that of the lighter piglets (Han et al. 2017). These results suggested that the host and environmental factors may play important roles in the formation of early microbial community structures in pigs.

In conclusion, the present results provided novel information of RFI-associated fecal bacterial profiles in Duroc pigs at early growth period, suggesting that the microbiota has a possible link with porcine FE. Importantly, the *Clostridiales* and *Bacteroidales*, such as *g_1_68*, *g_norank_f_p_2534_18B5*, were found to be potential early life predictive biomarkers for high FE. Predictive functional analysis also indicated that fecal microbes of the high FE pigs may have a high level of utilize dietary protein. Besides, our results indicated that the composition of fecal bacterial community was related to some host factors, especially litter size and parity. Although, as of now, more studies are required to clarify the relationship between the intestinal microbiota at a growing stage and FE at a mature stage pig, these results may provide insights into understanding the host-microbe interactions occurring in the early-life pig intestine and will be helpful for the assisted early selection of porcine FE.

## Supplementary information

**Additional file 1.** Characteristics of the pigs used in this study.

## Data Availability

The sequencing raw data in this study was deposited in NCBI Sequence Read Archive (SRA) under accession number PRJNA593419.

## Abbreviations

RFI: Residual feed intake
FE: Feed efficiency
KEGG: Kyoto Encyclopedia of Genes and Genomes
RDA: Redundancy analysis
DFI: Daily feed intake
BW: Body weights
ADFI: Average daily feed intake
ADG: Average daily gain
FCR: Feed conversion ratio
BF: Backfat
PCR: Polymerase chain reaction
QIIME: Quantitative Insights Into Microbial Ecology
OTUs: Operational taxonomic units
PCA: Principal component analysis
LEfSe: Linear discriminant analysis effect size
PICRUSt: Phylogenetic investigation of community by reconstruction of unobserved States
SCFA: Short chain fatty acids
